# High Resolution Magic Angle Spinning Proton NMR Study of Alzheimer’s Disease with Mouse Models

**DOI:** 10.3390/metabo12030253

**Published:** 2022-03-17

**Authors:** Mark V. Füzesi, Isabella H. Muti, Yannick Berker, Wei Li, Joseph Sun, Piet Habbel, Johannes Nowak, Zhongcong Xie, Leo L. Cheng, Yiying Zhang

**Affiliations:** 1 Department of Pathology, Harvard Medical School, Massachusetts General Hospital, Boston, MA 02115, USA; mfuzesi@mgh.harvard.edu (M.V.F.); imuti@mgh.harvard.edu (I.H.M.); jsun33@mgh.harvard.edu (J.S.); 2 Hopp Children’s Cancer Center Heidelberg (KiTZ), 69120 Heidelberg, Germany; yannick.berker@kitz-heidelberg.de; 3 Clinical Cooperation Unit Pediatric Oncology, German Cancer Research Center (DKFZ), German Cancer Consortium (DKTK), 69120 Heidelberg, Germany; 4 Department of Anesthesia, Critical Care and Pain Medicine, Harvard Medical School, Massachusetts General Hospital, Boston, MA 02115, USA; li.wei@mgh.harvard.edu (W.L.); zxie@mgh.harvard.edu (Z.X.); 5 Department of Medical Oncology, Haematology and Tumour Immunology, Charité—University Medicine Berlin, 10117 Berlin, Germany; phabbel@yahoo.de; 6 Radiology Gotha, SRH Poliklinik Gera, 99867 Gotha, Germany; johannes.nowak@yahoo.de; 7 Departments of Radiology and Pathology, Harvard Medical School, Massachusetts General Hospital, Boston, MA 02115, USA

**Keywords:** Alzheimer’s disease, mouse model, metabolomics, nuclear magnetic resonance spectroscopy

## Abstract

Alzheimer’s disease (AD) is a crippling condition that affects millions of elderly adults each year, yet there remains a serious need for improved methods of diagnosis. Metabolomic analysis has been proposed as a potential methodology to better investigate and understand the progression of this disease; however, studies of human brain tissue metabolomics are challenging, due to sample limitations and ethical considerations. Comprehensive comparisons of imaging measurements in animal models to identify similarities and differences between aging- and AD-associated metabolic changes should thus be tested and validated for future human non-invasive studies. In this paper, we present the results of our highresolution magic angle spinning (HRMAS) nuclear magnetic resonance (NMR) studies of AD and wild-type (WT) mouse models, based on animal age, brain regions, including cortex vs. hippocampus, and disease status. Our findings suggest the ability of HRMAS NMR to differentiate between AD and WT mice using brain metabolomics, which potentially can be implemented in in vivo evaluations.

## 1. Introduction

The National Institute on Aging (NIA) estimates that more than 6 million Americans now suffer from Alzheimer’s disease (AD), a complex neurodegenerative disorder that is the third-leading cause of death for older people, after heart disease and cancer [1]. The impact of the disease, however, is far greater, as AD also affects the lives of those caring for patients, as they struggle with progressive incapacitation. At present, AD is defined by the presence of amyloid-beta (Aβ) and tau protein aggregates in the brain and is driven by multiple pathophysiological processes, from proteostatic abnormalities to inflammation, vascular disease, and metabolic dysfunction [2]. Nonetheless, genetic, environmental, and life factors strongly contribute to its emergence and progression.

From AD’s discovery in 1906 to its presentations in today’s neurology clinics, AD diagnoses have relied on clinical evaluations of cognition, rather than evaluations of the disease itself. Major advances in imaging and biofluid biomarkers have supported the identification of Aβ and tau pathology, but the degree to which these pathologies’ presence leads to symptoms and progression, or to the biological factors driving their disease, varies considerably. Of note and more worrisome, autopsy is required for a definitive AD diagnosis through pathology examinations of brain tissue [3], and no definitive non-invasive examination is yet able to diagnose and characterize AD during a patient’s life. Furthermore, although AD-associated metabolic changes in the brain and other organs may assist the diagnosis of the disease, their metabolic concentrations can vary drastically under differing tissue preservation conditions after death, given the complexity of ex vivo metabolic evaluations (genetics or pathology measurements are less affected by tissue preservation). This variation challenges the direct use of human brain tissues to assist the discovery of aging- and AD-associated metabolic changes [4]. This complexity can be mitigated by controlled animal experiments with rigorously designed procedures.

To better understand the disease and, more importantly, to probe into AD through invasive and non-invasive evaluations, various AD mouse models have been developed since the 1990s [5,6]. The initially developed AD models focused on brain pathological hallmarks of the disease, and particularly on the aberrant accumulation of the peptide Aβ, which aggregates into plaques, and the microtubule-associated protein tau that forms tangles [7]. To achieve Aβ depositions in the brain, amyloid precursor protein (APP) models have been derived, both with and without APP overexpression; the latter has better resembled human conditions [8]. Models that produce humanized tau have also been developed [9]. In addition, to better represent the sporadic and late-onset characteristics seen in human disease, models incorporating the ε4 variant of the APOE gene were created [10]. With these models, disease-affected physiological and pathological changes in different brain regions, as well as other peripheral organs, can be analyzed using genomics, proteomics, and metabolomics platforms.

Metabolomics reflecting on-going metabolic activities can provide an instant “snap-shot” of disease conditions. Metabolomic investigations of AD have primarily concentrated on human patients and murine models. A few exceptions have ventured into such areas as analyses of cell lines [11,12] and *C. elegans* [13], as well as measurements of CSF in a rabbit AD model of high-cholesterol diet [14]. Studies using FTIR and Raman spectroscopy [15] have also been reported; however, the major methodologies used in AD metabolomic investigations are mass spectrometry (MS) and NMR. Studies of biological samples from human patients and transgenic animal models have been conducted with blood, various brain regions (hippocampus, cortex, cerebellum, striatum, olfactory bulbs, etc.), and other peripheral organs (liver, kidney, spleen, thymus, etc.) [16], as well as body fluids [17], such as human saliva [18,19,20,21], using technology developments that have ranged from amino acids profiling [22] to high-throughput metabolite fingerprints [23]. Most human MS studies have concentrated on blood serum and plasma [24,25,26,27] and CSF [28,29,30], particularly with biological samples collected from various clinical cohorts [31,32,33,34,35,36]. These multi-clinical cohort studies have relied on comparative analyses to differentiate among different brain regions [37] or between brain tissue and serum [35] or CSF [38], to establish serum metabolomic differences between AD and other neurological diseases [39], and to characterize incident dementia [40]. Associations between AD pathologies and metabolisms of polyamine [25,41], L-arginine [25], bile acids [32,33], unsaturated fatty acid [42] metabolisms, lipidomics [31], along with metabolomic comparisons of AD vs. normal aging [43,44] have been reported. High-resolution magic angle spinning (HRMAS) NMR can be utilized to study minute amounts (mg or μL) of intact tissues with a high spectral resolution that can identify metabolites as well as their relative alterations due to the relevant disease processes [45,46]. Recognizing the importance of metabolic changes in the development and progression of AD, as well as the difficulty of accurately quantifying metabolites with human brain specimens due to metabolic degradation after death, we designed this project to analyze animal AD models under rigorous experimental controls. Here, we report the metabolomics alterations observed from HRMAS NMR studies of AD and wild-type (WT) mouse models, based on animal age among females, brain regions for 9-month animals of both males and females, including cortex vs. hippocampus, and disease status.

## 2. Results

### 2.1. HRMAS NMR and Metabolomics

**Figure 1:**
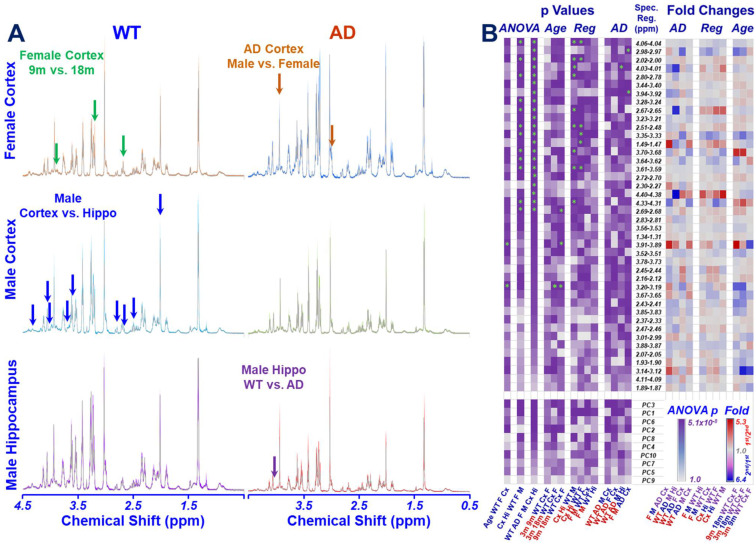
Intact Tissue HRMAS NMR Results of Mouse Brain Regions. (**A**). Examples of group-averaged spectra measured from WT and AD mice, with group standard deviations plotted as color shades, and with examples of statistically significant spectral regions after FDR corrections labeled with arrows. Hippo, hippocampus; m, month. (**B**). Heatmaps of *p*-values and fold changes measured between two tested groups (labeled in red on the horizontal axis). Reg, region; Spec, spectral; F, female; M, male; Cx, cortex; Hi, hippocampus; PC, principal component. *p*-values were compared between two groups labeled in red below the map, with green stars indicating significantly different regions after FDR corrections. Fold changes calculated using a red group (1st) over a blue group (2nd) are presented in the red scheme, whereas those using a blue group over a red group, the blue scheme.

High resolution proton NMR spectra of mouse brain tissue can be obtained using the HRMAS method, as shown in Figure 1A, where examples of group-averaged spectra measured from 9-month WT and AD of male and female animals are compared for cortex and hippocampus. Significant spectral regions (after false discovery rate (FDR) corrections) are labeled with arrows in the figure. Data obtained from these spectral analyses of the 42 identified spectral regions are summarized in Figure 1B, including (1) ANOVA results evaluated for group comparisons according to age, brain regions, and AD status, and (2) *t*-test results for binary comparisons between relevant groups, calculated from both individual spectral regions and individual principal components (PCs) from principal component analysis (PCA) as metabolomics profiles for males and females, respectively. Fold changes for the corresponding spectral regions measured between these relevant bi-groups are also presented in Figure 1B.

**Figure 2:**
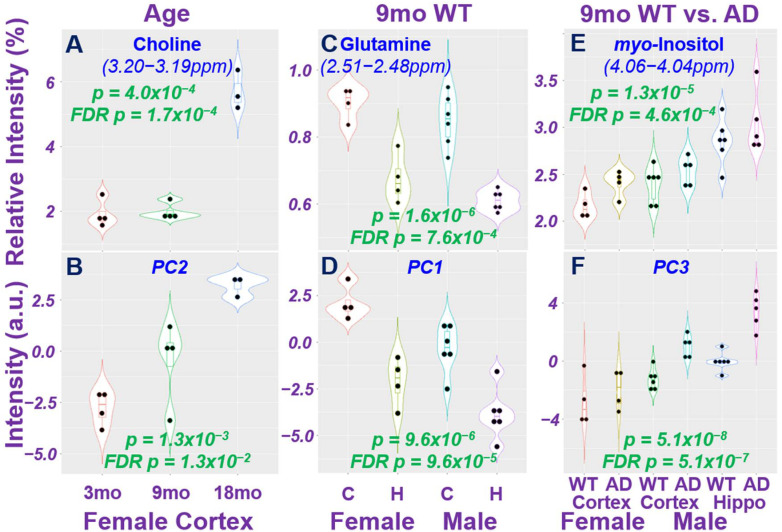
Differentiations of Animal Age, Brain Region, and AD Status using Individual Spectral Regions, and Principal Component as Metabolomics Profiles. Both original *p*-values and *p*-values after FDR corrections are presented in each plot. Mo, month; C, cortex; H, hippocampus; a.u., arbitrary unit.

Examples of statistically significant ANOVA results from Figure 1B are shown in Figure 2 for group comparisons according to age, brain regions, and AD status measured from either a single spectral region or a single PC of a metabolomics profile.

### 2.2. Age Difference among Females and Brain Regional Differences for 9-Month Male and Female Wild-Type Animals

**Figure 3:**
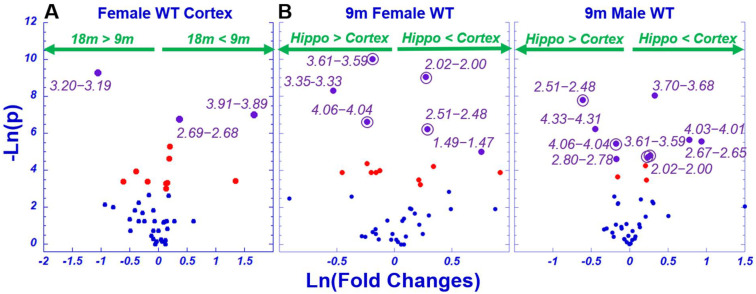
Differentiations of WT Animal Age and Brain Region with Individual Spectral Regions According to *p*-values and Fold Changes. (**A**) Age differentiations for female cortex; (**B**) cortex and hippocampus differentiations for female and male animals. All the analyzed 42 spectral regions are presented in plots, in which blue indicates insignificant points, red indicates significant original *p*-values, and purple indicates significant regions after FDR corrections. Regions labeled in purple with circles indicate common FDR significant regions seen in both males and females. Hippo, hippocampus; m, months.

Evaluations of AD-associated metabolomics alterations rely on accurate determinations of baseline metabolomics status measured from WT animal groups based on age and sex. In the current study, we evaluated the brain regions of WT animals of different ages, including cortex and hippocampus for 9-month males and females, as summarized in Figure 1B. Examples of these observed differences are shown in Figure 3A as fold changes vs. *p*-values of age differences for female mice, and of brain region differences for both males and females in Figure 3B. In these figures, significantly different regions (above −ln(0.05) = 2.996) are presented in red, and regions determined to be significant after FDR corrections are indicated in purple, including 3.20–3.19, 3.91–3.89, and 2.69–2.68 in Figure 3A. Figure 3B further indicates that, after FDR corrections, four spectral regions, 4.06–4.04, 3.61–3.59, 2.51–2.48, and 2.02–2.00, presented significant differences for both sexes, although the regions 3.61–3.59 and 2.51–2.48 indicate opposite directions. Overall, among 42 analyzed spectral regions, 14 presented FDR-verified significances in identifying these baseline differences.

### 2.3. AD-Associated Metabolomics Differences for 9-Month Animals

**Figure 4:**
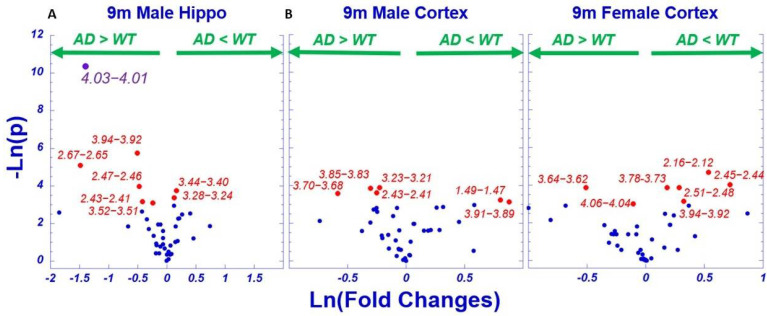
Differentiations of AD from WT for Animal Brain Regions using Individual Spectral Regions According to *p*-values and Fold Changes. (**A**) Hippocampus of male, and (**B**) cortex of female and male animals. Plot color scheme follows that used in Figure 3.

Following the analytic paradigm demonstrated in Figure 3, differentiations between AD and WT for different brain regions, cortex for both males and females, and hippocampus for male animals are examined in Figure 4. Among the 42 regions, 19 regions presented potential differentiations, but after FDR corrections, only the region 4.03–4.01 demonstrated significance for hippocampus tissues in male animals. Nevertheless, among these 19 regions, seven of them overlapped with significant baseline regions in Figure 3 after FDR corrections.

**Figure 5:**
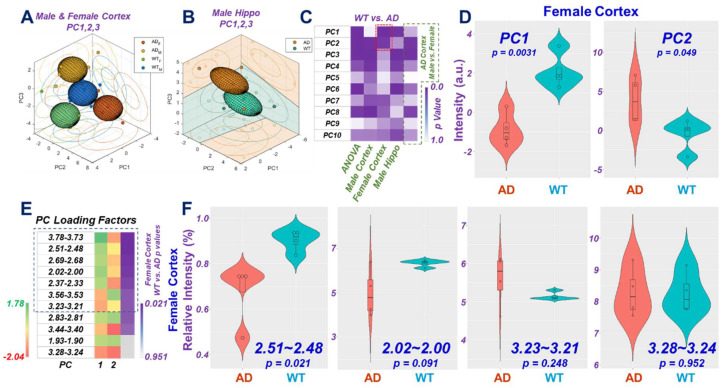
Differentiations of AD from WT using Tissue metabolomics Profiles Measured from Different Brain Regions. Three-dimensional plots of PC1, PC2, and PC3, for (**A**) cortex of female and male and (**B**) hippocampus of male animals. The 3D ellipsoids generated from these PCs that cover the volume of 3D Mahalanobis distance ≤1 (one standard deviation from the centroid along each axis) from the class means for AD and WT groups, according to the covariance matrix of all class-mean-corrected samples. Group separations are clearly presented in these plots; (**C**) *p*-values for differentiations between AD and WT for the tested groups; (**D**) examples of differentiating AD from WT by using PC1 and PC2 calculated from female cortex. A revisal of correlations is seen with these two PCs; (**E**) 11 of the 42 analyzed female cortex spectral regions appeared as common regions in either the top or the bottom 50% of major contributing spectral regions towards the determinations of PC1 and PC2. These regions are listed (in (**E**)) in ascending order according to the *p*-values of the spectral regions in comparing AD from WT (coded in purple). Green color indicates the region’s positive contribution towards the final PC values, and red color a negative contribution; (**F**) examples of individual regions seen in (**E**), where small *p*-values corresponding to large color differences between green and red (cf. 2.51~2.48) that translate to polar contributions to the PC values, and large *p*-values corresponding to small color differences (cf. 3.28~3.240), contribute non-polarly to the PC values.

In addition to the analyses of individual spectral regions, we further tested the capabilities of PC-represented metabolomics profiles in differentiating AD from WT for the measured male and female animal groups. The 3-D plots for PCs of cortex and hippocampus are illustrated in Figure 5A,B, respectively, and clearly demonstrate the group separations achievable with combined considerations of PC1, 2, and 3. The *p*-values for bi-group comparisons with individual PCs are shown in Figure 5C.

Differentiations of AD from WT using PC1 and PC2 measured for cortex samples of female mice are shown in Figure 5D. The contribution of a specific spectral region towards the calculated PC depends on its loading coefficient, determined through PCA, as well as the mean and the standard deviation calculated for that region with all analyzed individual samples. The resulting overall loading factor for the spectral region is the product of the loading coefficient and the ratio of the mean over the standard deviation. Among the 42 overall loading factors for the 42 regions, we examined the top 50% of positively contributing regions and the bottom 50% of negatively contributing regions for PC1 and PC2, as shown in Figure 5E. Figure 5E presents only regions that appear in both PCs as either the top or bottom 50% of major contributing regions and are ordered according to *p*-values from the *t*-test of AD vs. WT for the cortex of female mice. Of note, among these 11 regions, the top 7 indicated opposite relationships between PC1 and PC2 that agree with the opposite direction presented in Figure 5D. Examples of these 11 regions and their abilities to differentiate AD vs. WT are shown in Figure 5F.

## 3. Discussion

To better understand human AD without directly accessing human brain tissue during life, a variety of animal models have been developed for laboratory tests and studies. The initial models focused on pathological hallmarks of the disease in the brain, particularly on the abnormal accumulation of the peptide Aβ, which aggregates into plaques, and the microtubule-associated protein tau that forms tangles [7]. To achieve Aβ depositions in the brain, amyloid precursor protein (APP) models have been derived, both with and without APP overexpression; the latter has better resembled human conditions [8]. Models that produce humanized tau have also been developed [9]. In addition, to better represent the sporadic and late-onset characteristics seen in human disease, models incorporating the ɛ4 variant of the APOE gene were created [10]. In the current study, we tested 5xFAD transgenic AD and C57/BL6 WT mice and used HRMAS proton NMR to measure brain metabolomics.

Immediately after the invention of HRMAS NMR to study intact brain tissue obtained from neurodegenerative Pick disease [45], we applied HRMAS NMR to human AD brain tissues and, for the first time, quantified a linear correlation between amounts of surviving neurons in human brains and concentrations of neuronal metabolite n-acetyl-aspartate (NAA) [47]. This method has the advantages of measuring the microgram scale of tissues without sample pre-processing and preserving tissue pathological structures for post-NMR pathological evaluations. It been used to study animal age-, sex-, and brain region-specific GABA levels [48] and other metabolic alterations [49]; mouse models under treatments of fingolimod [50], methylene blue [51], or dietary supplementation [52]; and blood metabolomic signatures of AD mice [53].

In our current study, we identified a number of metabolites that show FDR-corrected, statistically significant differences between animal groups; for instance, the spectral regions 3.20–3.19 of choline (Chol) and 3.91–3.89 of creatine (Cr) and Glycero-phosphocholine (GPC) from the cortex of female mice can differentiate animal age. Both Chol and Cr have been previously implicated in Alzheimer’s disease research. A 2021 review of MRI investigations of AD noted that studies have shown a decreased N-acetylaspartic/creatine ratio and increased myoinositol/creatine ratio in Alzheimer’s patients, compared to healthy controls [54]. Further, choline has been demonstrated to affect AD pathology. A study published in 2019 found that lifelong dietary supplementation with choline significantly reduced AD symptoms. Choline plays an important role in the central nervous system since it is a precursor for acetylcholine, a key regulatory molecule [55]. Due to the relationship between age and AD onset, identifying metabolites that correlate with age may also indicate the likelihood of disease. Further, significant metabolites have been found to differentiate brain metabolisms of the cortex and hippocampus for both male and female animals, including myo-inositol (4.06–4.04) and NAA (2.02–2.00), as demonstrated in Figure 3.

By measuring brain tissues obtained from the cortex and hippocampus of AD and WT animals, we have demonstrated that the power of differentiating AD from WT can be greatly increased using unsupervised metabolomics profiles obtained from PCA analysis of metabolites represented by individual spectral regions. By comparing the AD-WT differentiating directions of the PCs, such as PC1 and PC2 in Figure 5D, the loading factors from the major contributing spectral regions that are common for both PC1 and PC2 (Figure 5E), and the differentiating directions of these individual spectral regions (Figure 5F), the PC-represented metabolomics profiles can be interpreted through various metabolic pathways. These pathways include the top seven regions indicated in Figure 5E, representing glutamate (Glu), glutamine (Gln), NAA, glycine (Gly), Phosphoryl choline (PChol), and GPC, that contributed to PC1 and PC2 in different directions. For the other four spectral regions at the bottom of Figure 4E, although they were common for PC1 and PC2, they contributed to the same direction, which does not agree with the direction switching seen in Figure 5D, and individually, they showed no capability for AD-WT differentiation, as shown in Figure 5F.

The current NMR study of mouse models has a number of limitations. Although multiple animal groups of AD and WT mice have been evaluated, there are still many missing comparison groups, and for many of the tested groups, the number of animals is the minimum for data analysis. In addition, although we observed statistically significant spectral region differences between animal groups and attempted to associate them with metabolites, these assignments were based only on their chemical shift values, and no corroborations were attempted with other analytical approaches.

## 4. Materials and Methods

### 4.1. Animal Models

The animal protocol was approved by the Massachusetts General Hospital (Boston, MA, USA) Standing Committee on the Use of Animals in Research and Teaching (Protocol number: 2006N000219). All experiments were performed in accordance with the National Institutes of Health guidelines and regulations. Efforts were made to minimize the number of animals used. Mice had a 12:12 h light–dark cycle (lights on 7:00 am) with food and water available ad libitum. The mice were housed in a non-germ-free facility, mimicking real world conditions. AD Tg mice (B6SJL-Tg(APPSwFlLon, PSEN1*M146L*L286V)6799Vas/Mmjax, Stock No. 34848-JAX, Jackson Lab, Bar Harbor, ME, USA) and WT mice (C57BL/6J, Jackson Lab) were used in the study.

### 4.2. Harvest of Mice Brain Tissues

We harvested both the cortex and hippocampus from WT female mice at 3, 9, and 18 months (N = 4, 4, 3, respectively); the cortex from AD female mice at 9 months (N = 4); and the cortex and hippocampus from WT and AD male mice at 9 months (N = 6, 5, respectively). Missing subgroups were due to a lack of availability. We followed the detailed procedure described previously [56,57]. The mice were sacrificed by carbon dioxide euthanasia at room temperature. The brain was removed rapidly on dry ice, and the cortex and hippocampus were dissected out and frozen in liquid nitrogen for subsequent use in the NMR studies. The whole process was completed within 5–10 min [56,57].

### 4.3. HRMAS NMR

All NMR measurements were conducted on a Bruker AVANCE III HD 600 MHz spectrometer (Bruker BioSpin, Billerica, MA, USA). Before measurements, frozen brain tissue samples were weighed (~10 mg) with 2.5 μL of D_2_O and loaded into the 4 mm rotor, using a 12 μL Kel-f insert. HRMAS NMR data were collected at 4 °C with a spinning rate of 3600 Hz and with a rotor synchronized CPMG method. The other spectral conditions included: 5 s recycle time, 100 CPMG πpulses with a total mixing time of 55.56 ms, 16 K data points with a total acquisition time of 0.85 s, and a spectral width of 16 ppm.

### 4.4. Data Analyses

Spectra analysis was conducted off-line with Bruker Topspin 3.6.2 (Bruker BioSpin, Billerica, MA, USA). The spectral processing procedure included: 0.5 Hz line-broadening, one-time zero-fill to 32 K data points, Fourier transformation, automatic and manual phasing, baseline correction, chemical shift calibration according to the up-field peak of lactate doublets at 1.32 ppm, and resonance peak curve-fitting for complete deconvolutions. Within the analyzed 4.5 to 0.5 ppm spectral region, the total spectral intensity was used to normalize the measured and deconvoluted spectral peak intensities. From these valid deconvoluted peaks, using 95% of all measured individual samples having non-zero values as the threshold, 42 spectral regions were identified for further statistical analyses. Details of these regions and their potential major contributing metabolites are presented in Supplementary Table S1 based on published reference chemical shift values [58]. For further analysis, the intensity for each of these 42 spectral regions was normalized to the summed intensity of all 42 regions. In this report, we analyze them as spectral regions, rather than metabolites, for multiple metabolites may contribute to a single region, and vice versa, each metabolite may present in different regions. Nevertheless, we discuss their potential metabolic implications when appropriate.

Statistical analyses on these identified regions were carried out on JMP from SAS Institute (Cary, NC, USA), including univariate analysis according to Student’s *t*-test, or ANOVA (for normally distributed and equal variance data), Welch test (for normally distributed and unequal variance data), and Wilcoxon/Kruskal–Wallis test (KWW, for non-normally distributed data), as well as unsupervised multivariate principal component analyses. Following PCA, individual principal components were analyzed using the same protocol as outlined above for the individual spectral regions. In the presentation, these final analytic results, whether from Student’s *t*-test, ANOVA, Welch test, or KWW based on data structure, were all referred to as either *t*-test, for a two-grope comparison, or ANOVA, for comparisons of more than two groups. Both *p*-values before and after false discovery rate corrections are presented and indicated where appropriate.

## 5. Conclusions

Recognizing the need to understand age- and AD-associated brain metabolism and the uncertainty associated with ex vivo analyses of human brain tissues, we utilized AD mouse models to demonstrate the ability of HRMAS NMR technology to differentiate diseased from wild-type brain tissue. These initial results suggest the potential for future translational research with important clinical implications, including comprehensive comparisons of in vivo and ex vivo imaging measurements in animal models to identify similarities and differences between aging- and AD-associated metabolic changes. Establishing connections between in vivo imaging and ex vivo brain tissue and serum measurements will enable us to design in vivo imaging and blood serum evaluation protocols to test age- and AD-associated metabolomic changes in humans, aimed at non-invasively detecting AD onset, development, and progression, monitoring the effects of potential future therapies, and understanding AD metabolic mechanisms.

## Data Availability

Upon publication, data can be found at the MGH A. A. Martinos Center for Biomedical Imaging website.

## Supplementary Materials

The following supporting information can be downloaded at: https://www.mdpi.com/article/10.3390/metabo12030253/s1, Table S1: Major contributing metabolites for identified spectral regions.
