# Supplementary material for: High Resolution Magic Angle Spinning Proton NMR Study of Alzheimer’s Disease with Mouse Models
Source: Metabolites. 2022 Mar 17;12(3):253. doi: 10.3390/metabo12030253 (PMC8952313; doi:10.3390/metabo12030253)
Supplement: Supplementary file 1 [file metabolites-12-00253-s001.zip › metabolites-1631466-supplementary.pdf]

Table S1. Major contributing metabolites for identified spectral regions. Abbreviations: adenosine triphosphate (ATP), N-acetylaspartate (NAA), glycerophosphocholine (GPC), lactate (Lac), choline (Chol), myo-inositol (m-Ino), tyrosine (Tyr), serine (Ser), phosphocreatine (PCr), creatine (Cr), glucose (Glc), alanine (Ala), glutathione (GSH), glutamine (Gln), glutamate (Glu), phosphoryl choline (Pchol), valine (Val), glycine (Gly), taurine (Tau), scyllo-inositol (s-Ino), phenylalanine (Phe), histidine (His), aspartate (Asp), pyruvate (Pyr), gamma-aminobutyric acid (GABA), N-acetylaspartylglutamic acid (NAAG), acetone (Acet), To Be Determined (TBD).

4.40-4.38	*ATP, NAA*
4.33-4.31	*GPC*
4.11-4.09	*Lac*
4.06-4.04	*Chol, m-Ino, Tyr*
4.03-4.01	*TBD*
3.94-3.92	*Ser, PCr, Tyr*
3.91-3.89	*Cr, GPC*
3.88-3.87	*Glc*
3.85-3.83	*GPC, Glc, Ser,*
3.78-3.73	*Ala, GSH, Glycerol, Glc, Gln, Glu*
3.70-3.68	*Glc*
3.67-3.65	*GPC*
3.64-3.62	*Glycerol, Pchol*
3.61-3.59	*GPC, m-Ino, Val*
3.56-3.53	*Glycerol, Gly*
3.52-3.51	*Glc, m-Ino,*
3.44-3.40	*Tau, Glc*
3.35-3.33	*s-Ino*
3.28-3.24	*m-Ino, Phe, Tau,*
3.23-3.21	*Glc, His, Phosphoryl ethanolamine, GPC, Pchol*
3.20-3.19	*Chol, Homocarnosine, Tyr*
3.14-3.12	*His*
3.01-2.99	*GABA, Homocarnosine, Histamine*
2.98-2.97	*Histamine, GSH*
2.83-2.81	*TBD*
2.80-2.78	*Asp*
2.72-2.70	*NAAG*
2.69-2.68	*TBD*
2.67-2.65	*NAA, Asp*
2.51-2.48	*GSH, NAA*
2.47-2.46	*TBD*
2.45-2.44	*Gln*
2.43-2.41	*Gln*
2.37-2.33	*Homocarnosine, Pyr, Glu*
2.30-2.27	*GABA*

2.16-2.12	*GSH, Gln, Glu*
2.07-2.05	*NAAG*
2.02-2.00	*NAA*
1.93-1.90	*Acet*
1.89-1.87	*GABA, Homocarnosine, NAAG*
1.49-1.47	*Ala*
1.34-1.31	*Lac*
